# Phenotypic spectrum and antialbuminuric response to angiotensin converting enzyme inhibitor and angiotensin receptor blocker therapy in pediatric Dent disease

**DOI:** 10.1002/mgg3.1306

**Published:** 2020-06-03

**Authors:** Haiyue Deng, Yanqin Zhang, Huijie Xiao, Yong Yao, Hongwen Zhang, Xiaoyu Liu, Baige Su, Na Guan, Xuhui Zhong, Suxia Wang, Jie Ding, Fang Wang

**Affiliations:** ^1^ Department of Pediatrics Peking University First Hospital Beijing China

**Keywords:** albuminuria, *CLCN5*, Dent disease, *OCR*L

## Abstract

**Background:**

To characterize the phenotypic spectrum and assess the antialbuminuric response to angiotensin converting enzyme (ACE) inhibitor and/or angiotensin receptor blocker (ARB) therapy in a cohort of children with Dent disease.

**Methods:**

The patients’ clinical findings, renal biopsy results, genetic and follow‐up data were analyzed retrospectively. Mutations in *CLCN5* or *OCRL* were detected by next‐generation sequencing or Sanger sequencing.

**Results:**

Of 31 Dent disease boys, 24 carried *CLCN5* and 7 carried *OCRL* mutations. Low molecular weight proteinuria and albuminuria were detected in all cases. Nephrotic‐range proteinuria and severe albuminuria were identified in 52% and 62% of cases, respectively; by 7 years of age, 6 patients had hematuria and nephrotic‐range proteinuria, and 7 patients had hematuria and moderate to severe albuminuria. In addition to disease‐related renal features, patients with Dent‐1 disease also presented with congenital cataract (1/9) and developmental delay (2/7). Seventeen of 31 patients underwent renal biopsy. Glomerular changes included mild glomerular lesions, mesangial proliferative glomerulonephritis and focal segmental glomerular sclerosis. Thirteen of the 31 patients had follow‐up records and received ACE inhibitor and/or ARB treatment for more than 3 months. After a median 1.7 (range 0.3–8.5) years of treatment, a reduction in the urinary microalbumin‐to‐creatinine ratio was observed in 54% of children.

**Conclusions:**

Hematuria with nephrotic‐range proteinuria or moderate to severe albuminuria was common in Dent disease patients. Extrarenal manifestations were observed in Dent‐1 patients, which extends the phenotypic spectrum. In addition, ACE inhibitors and ARBs are well tolerated, and they are partially effective in controlling albuminuria.

## Graphical abstract

- Hematuria with nephrotic‐range proteinuria or moderate to severe albuminuria was common in Dent disease patients.
- Patients with Dent‐1 disease may also present with congenital cataract and developmental delay.
- The percentage of urinary albumin in the total protein ranged from 24.2% to 47.6%; it was usually smaller than that of LMW proteinuria. ACE inhibitors and ARBs are partially effective in controlling albuminuria and they are well tolerated.

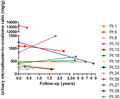

## 1 INTRODUCTION

Dent disease is a rare X‐linked recessive chronic kidney disease (CKD). Proximal renal tubular dysfunction, caused by mutations in the *CLCN5* (OMIM# 300008, classified as Dent‐1 disease, accounting for approximately 60%) or *OCR*L (OMIM# 300535, classified as Dent‐2 disease, accounting for 15%) genes, leads to the clinical manifestations of this disease (Zhang, Fang, Xu, & Shen, 2017). Dent disease is characterized by low molecular weight (LMW) proteinuria, hypercalciuria, nephrocalcinosis, nephrolithiasis, and chronic renal failure (Lieske et al., 1993), and LMW proteinuria is the essential phenotype (Sekine et al., 2014). Affected males usually do not progress to end‐stage renal disease before the age of 30 years (Devuyst & Thakker, 2010). Since proteinuria is always the initial symptom, and since nephrotic‐level proteinuria can be detected in more than half of children with Dent disease (van Berkel, Ludwig, van Wijk, & Bokenkamp, 2017), it is not unusual for the disease to be misdiagnosed and mistreated (Wang et al., 2016). Therefore, understanding the phenotypic spectrum of Dent disease is helpful to further improve the reorganization of this disorder.

Recently, a study (Wang et al., 2016) showed that, in patients with Dent disease, glomerular pathology, including focal global glomerulosclerosis and foot process effacement, was common and was associated with renal function progression. As a principal biomarker for renal dysfunction, albuminuria increased as well as LMW proteins in the course of Dent disease (Kubo et al., 2016). These results indicated that angiotensin converting enzyme (ACE) inhibitors and angiotensin receptor blockers (ARBs) might be potential interventions for Dent disease. However, there is a scarcity of data for the effectiveness of these drugs on albuminuria in Dent disease.

The aim of the present study was to characterize the phenotypic spectrum and assess the antialbuminuric response to ACE inhibitor and/or ARB therapy in a Chinese cohort of children with Dent disease.

## 2 SUBJECTS AND METHODS

### 2.1 Ethical compliance

This study was performed in accordance with the Declaration of Helsinki, and the Ethical Committee of Peking University First Hospital approved this project.

### 2.2 Patients

**FIGURE 1:**
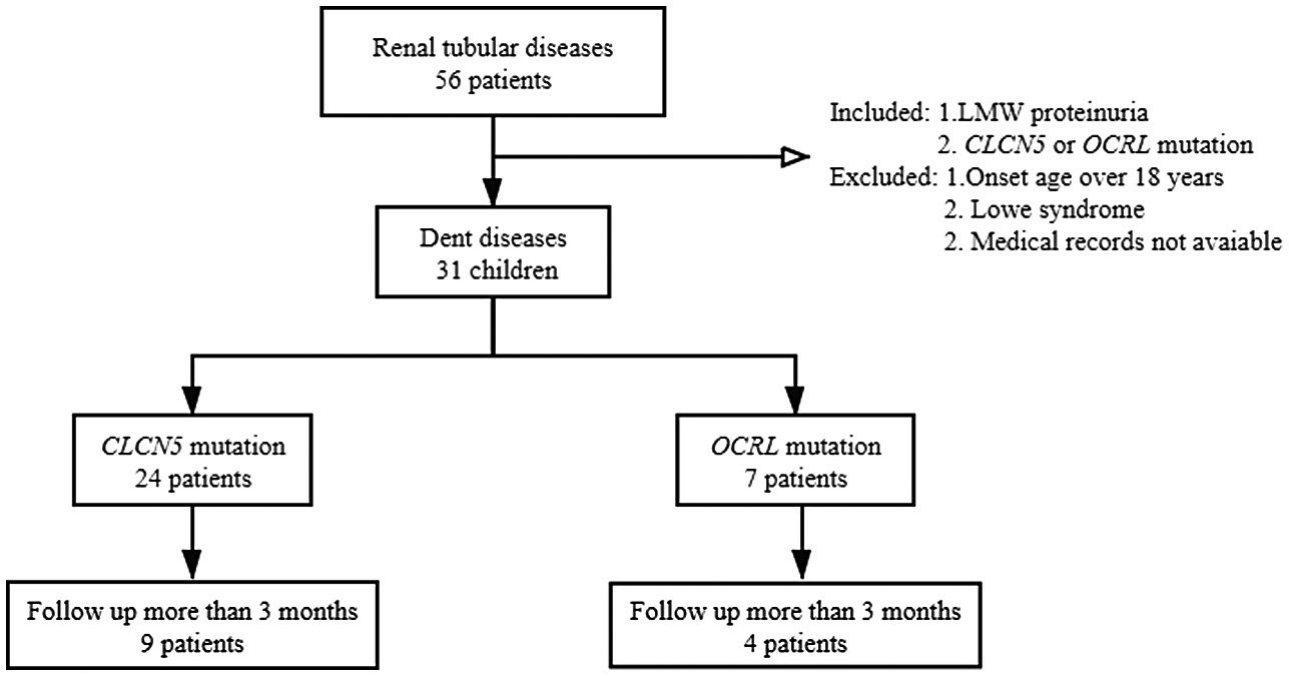
Flow diagram on detection of Dent disease from hereditary renal diseases registry database

Data for this study were collected retrospectively from our hereditary renal diseases registry database initiated in 2012. The following criteria were used to enroll patients: (1) LMW proteinuria; and (2) mutations in *CLCN5* or *OCRL* detected by next‐generation sequencing or Sanger sequencing. Patients were excluded if their age at onset of disease was over 18 years, if they were diagnosed with Lowe syndrome, or if their medical records were not available (Figure 1).

### 2.3 Research methods

Patient information, including sex, race, age, clinical findings (age at onset of disease, first symptoms, kidney and extrarenal features), renal biopsy results, genotypes and follow‐up data, were reviewed.

LMW proteinuria was defined as spot morning urine sample α1‐microglobulin (α1‐MG) 5 times above the upper limit of normal (Lieske et al., 1993). Mild, moderate and severe albuminuria referred to microalbumin‐to‐creatinine ratios (mg/g, ACR) <30, 30–300, and ≥300, respectively (Levin & Stevens, 2014). Nephrotic‐range proteinuria was defined as 24‐hr urine protein > 50 mg/kg. Hypercalciuria was defined as urinary calcium > 0.1 mmol/kg/d or a calcium‐to‐creatinine ratio above the 95th percentile of reference values in random urine (Lieske et al., 1993). Since the level of serum phosphate in children varies with age, an age‐based serum phosphate reference was used to diagnose hypophosphatemia (Ruppe, 1993). Nephrocalcinosis was diagnosed by renal ultrasound. The glomerular filtration rate (GFR) was estimated using 24‐hr endogenous creatinine clearance (Ccr) (National Kidney, 2002), and the Schwartz formula (Schwartz, Brion, & Spitzer, 1987) was used when 24‐hr Ccr was unavailable. Renal dysfunction was defined as a GFR below 75 ml/min/1.73 m^2^ (Pottel, Hoste, & Delanaye, 2015). Short stature was defined as height below the 3rd percentile of the reference for Chinese children. Intellectual disability and developmental delay were evaluated by Gesell developmental schedules, Wechsler intelligence scale or Raven intelligence test. Rickets was diagnosed by bone X‐ray or physical examination. When there were more than two measurements of corresponding parameters in a month, we calculated the mean values and used them for subsequent analysis.

Genomic DNA, extracted from peripheral blood leukocytes in all participants, was analyzed by next generation sequencing or Sanger sequencing. Whenever DNA from first‐degree relatives of the participants were available, segregation analysis was performed by Sanger sequencing. The interpretation of unpublished variants in *CLCN5* or *OCRL* was performed according to American College of Medical Genetics and Genomics (ACMG) guidelines (Richards et al., 2015).

## 3 RESULTS

As shown in Figure 1, a total of 31 male patients with Dent disease were included in this study. They were from 14 provinces in China and were predominantly of Han Chinese ethnicity (25/29), with the remainder being Hui, Manchu, Bai and Mongolian nationality.

All patients had a genetic diagnosis at the median age of 6.3 (range 1.7–16.8) years old. The median duration from disease onset to genetic diagnosis was 31.2 (range 2.4–187.2) months.

The different *CLCN5* and *OCRL* mutation categories detected in the present study are summarized in Table 1. Almost half of these mutations were not published previously. Table 2 lists the interpretation of these novel mutations according to ACMG guidelines. Among them, 13 variants were classified as pathogenic. Patients 7, 26 and 30 were highly suspected of Dent disease based on LMW proteinuria and hypercalciuria with or without nephrocalcinosis; thus, the novel variants identified in these three patients were classified as clinically pathogenic.

**TABLE 1:** Type of mutations in Dent disease cohort

	Types of mutations	Number of patients	Number of novel mutations
Dent‐1 disease (*CLCN5*)	Missense	5 (20.8%)	2
	Nonsense	8 (33.3%)	1
	Frame shift	9 (37.5%)	8
	Deletion	1 (4.2%)	1
	Splice	1 (4.2%)	1
	Total, *n* %	24	13
Dent‐2 disease (*OCRL*)	Missense	4 (57.1%)	2
	Nonsense	1 (14.3%)	1
	Frame shift	1 (14.3%)	0
	Deletion	1 (14.3%)	0
	Total, *n* %	7	3

**TABLE 2:** 16 *CLCN5* and *OCRL* novel mutations identified

Pt.ID	Gene	Chromosome position	Location	Nucleotide changes	Amino acid changes	Segregation	ACMG	
							Classify sequence variants	Interpretation
1	*CLCN5*	chrX:49840517	EX 7	c.483delA	p.Gly163Aspfs*45	maternal	PVS1 PM2 PM4 PP3 PP4	Pathogenic
3	*CLCN5*	chrX:49854931‐49854934	EX 13	c.1903_1906delAATG	p.Asn635Aspfs*20	maternal	PVS1 PM2 PM4 PP3 PP4	Pathogenic
4	*CLCN5*	chrX:49851383‐49851384	EX 11	c.1413_1414delTG	p.Cys471X	maternal	PVS1 PM2 PM4 PP1 PP3 PP4	Pathogenic
7	*CLCN5*	chrX:49853517	EX 12	c.1720A>G	p.Met574Val	maternal	PM1 PM2 PP3 PP4	Likely pathogenic
11	*CLCN5*	chrX:49850643	EX 10	c.940delT	p.Ser314Argfs*10	NA	PVS1 PM2 PM4 PP3 PP4	Pathogenic
13	*CLCN5*	chrX:49855003	EX 13	c.1975delC	p.Arg659Glyfs*7	maternal	PVS1 PM2 PM4 PP3 PP4	Pathogenic
16	*CLCN5*	chrX:49806925‐49856876	EX 4‐15^a^	c.17‐2451del	‐	NA	PVS1 PM1 PM2 PM4 PP4	Pathogenic
17	*CLCN5*	chrX:49850695‐49850696	EX 10	c.992‐993insAGTATTAT	p.Pro334fs*1	maternal	PVS1 PM2 PM4 PP3 PP4	Pathogenic
18	*CLCN5*	chrX:49850692	EX 10	c.989G>A	p.Gly330Asp	de novo	PS2 PM1 PM2 PM5 PP3 PP4	Pathogenic
19	*CLCN5*	chrX:49854917‐49854918	EX 13	c.1889‐1990delC	p.His631Thrfs*25	maternal	PVS1 PM2 PM4 PP3 PP4	Pathogenic
21	*CLCN5*	chrX:49854771	IVS12	c.1745‐2A＞G	Splice defect	maternal	PVS1 PM2 PP3 PP4	Pathogenic
22	*CLCN5*	chrX:49854779	EX 13	c.1751delT	p.Val584Glyfs*4	maternal	PVS1 PM2 PM4 PP3 PP4	Pathogenic
23	*CLCN5*	chrX:49851414	EX 11	c.1444delG	p.Gly483Vfs*21	maternal	PVS1 PM2 PM4 PP3 PP4	Pathogenic
26	*OCRL*	chrX:128722956	EX 22	c.2435T>C	p.Leu812Pro	maternal	PM1 PM2 PP4	Uncertain significance
30	*OCRL*	chrX:128696629	EX 12	c.1110C＞G	p.Cys370Trp	maternal	PM1 PM2 PP3 PP4	Likely pathogenic
31	*OCRL*	chrX:128691332	EX 5	c.269G＞A	p.Trp90X	maternal	PVS1 PM2 PP3 PP4	Pathogenic

Note

We login Human Gene Mutation Database (HGMD) to check novel mutations in July, 2019. Accession no: *CLCN5* (OMIM#300008)*,* NM_001127899.4; *OCRL* (OMIM#300535)*,* NM_000276.4; NA, not available.

^a^ Agarose gel electrophoresis showed that Patient 16 has a deletion from exon 3 to 15.

Of 31 patients, 22 patients (71%) were misdiagnosed. Among them, 19 patients received inappropriate steroid and/or immunosuppressive therapy, and genetic diagnosis was made at the median age of 6.9 (range 3.8–16.8) years old. The median duration from onset of disease to genetic diagnosis in these patients was 30 (range 2.4–187.2) months.

### 3.1 Characteristics of onset symptoms

The median age at disease onset was 3.7 (range 0.2–12.3) years old. Manifestations (including abnormal urine color, multiple urine foam, frequent micturition and edema) at disease onset were observed in 13 males (42%) at the median age of 4 (range 0.2–7.3) years, and 18 patients (58%) were asymptomatic; they were found accidentally for other reasons at the median age of 3.4 (range 0.3–12.3) years old.

### 3.2 Renal features

**FIGURE 2:**
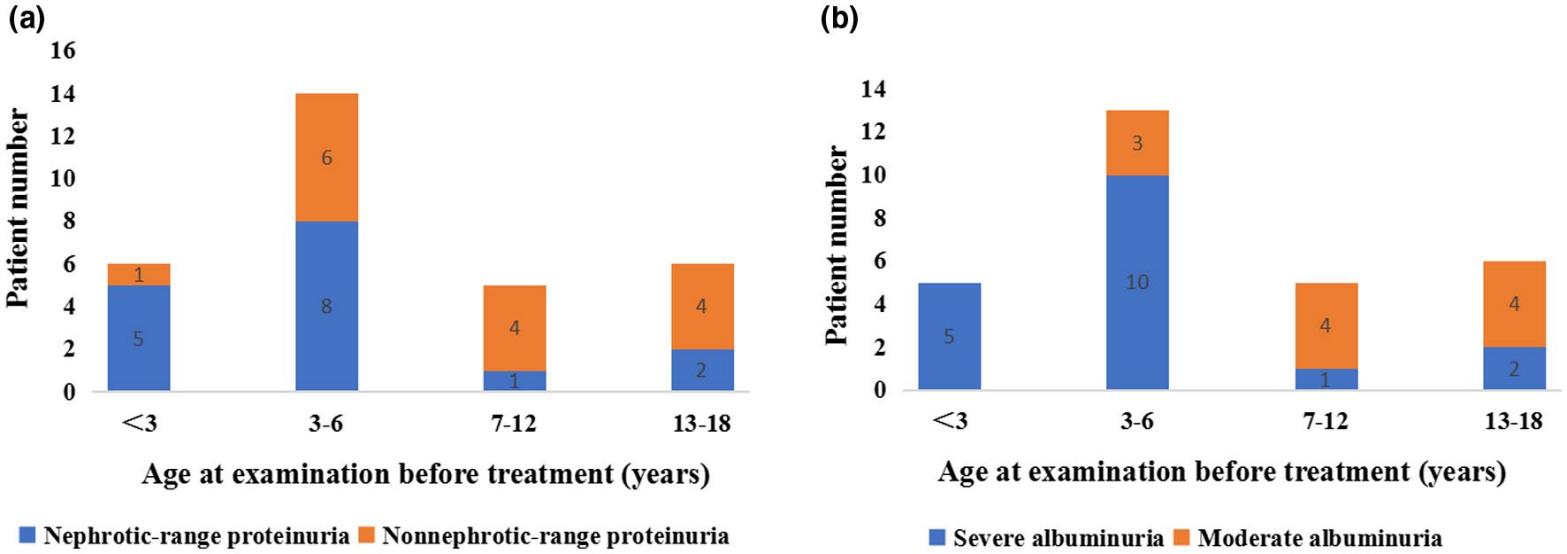
Proteinuria features observed at different ages. Probands are categorized by age undergoing relevant examinations before treatment. (a) Distribution of nephrotic range proteinuria (blue) and nonnephrotic range proteinuria (orange) at different ages. (b) Distribution of severe albuminuria (blue) and moderate proteinuria (orange) at different ages

A description of the renal manifestations before treatment is given in Table 3. LMW proteinuria and albuminuria were detected in all cases. The median percentage of urinary albumin detected by urine protein electrophoresis was 36% (range 24.2%–47.6%). Nephrotic‐range proteinuria and severe albuminuria were identified in 52% (16/31) and 62% (18/29) of patients, respectively. In patients younger than 7 years old, the percentages of nephrotic‐range proteinuria and severe albuminuria were 65% (13/20) and 83% (15/18), respectively. Notably, nephrotic‐range proteinuria and severe albuminuria were detected in 83% (5/6) and 100% (5/5) of patients younger than 3 years old, respectively (Figure 2). Microscopic hematuria was detected in 32% (10/31) of patients. Before the age of 7 years, 6 patients had hematuria and nephrotic‐range proteinuria, 7 patients had hematuria and moderate to severe albuminuria. Hypercalciuria was detected in 87% (27/31) of patients and occurred at all ages, whereas only 33% (9/27) presented with hematuria. Twenty‐seven patients underwent renal ultrasound, no nephrolithiasis was identified, whereas nephrocalcinosis was detected in 30% (8/27) of patients. There were only 15 patients who underwent urinary amino acid analysis, and all of them had aminoaciduria. Only four of 27 (15%) patients manifested abnormal GFRs. Among them, one patient (patient 13) presented with nephrotic‐level proteinuria (3.6 g/24 hr), bilateral renal cysts, and renal dysfunction (Ccr was 70 ml/min) at the age of 12.9 years. A half years later, he still manifested with nephrotic‐level proteinuria (3.02 g/24 hr) while his renal dysfunction progressed (Ccr was 48 ml/min). Bilateral renal cysts may lead to the patient's renal dysfunction. There were no significant differences in renal features between patients with Dent‐1 disease and Dent‐2 disease (Table 4).

**TABLE 3:** Renal features of patients with Dent disease

Pt.ID	Diagnosis	Age at examination before treatment/onset (years)	Nephrotic‐range proteinuria	Urinary α1‐MG/Cr (mg/g)	Percentage of LMWP	Percentag of urinary albumin	Urinary ACR (mg/g)	Hypercalciuria	Nephrocalcinosis (age, years)	Hematuria	Hypophosphatemia	Abnormal GFR
D‐1	Dent‐1	5.3/4.8	Yes	1,125.52	56.2	29	Severe	Yes	− (5.3)	Yes	No	No
D‐2	Dent‐1	1/0.8	Yes	1,184.68	51.4	40	Severe	Yes	+ (1)	No	No	No^a^
D‐3	Dent‐1	5.6/1	Yes	578.86	54.9	39.8	Severe	Yes	− (5.6)	No	No	No^a^
D‐4	Dent‐1	16.6/8	No	289.5	55.5	35.1	Moderate	No	− (16.6)	Yes	No	No
D‐5	Dent‐1	3.8/1.5	No	425.55	64.4	32.6	Moderate	Yes	− (3.8)	No	No	No
D‐6	Dent‐1	4.3/3.2	Yes	583.85	48.2	40.6	Severe	Yes	− (4.3)	No	No	No
D‐7	Dent‐1	15.4/4	No	456.3	50.1	36.1	Moderate	Yes	+ (15.4)	No	No	No
D‐8	Dent‐1	13.1/3	No	514.23	51.4	38.4	Moderate	Yes	+ (13.2)	Yes	No	No
D‐9	Dent‐1	2.1/2	Yes	1,350.45	51	38.1	Severe	Yes	NA	No	Yes	NA
D‐10	Dent‐1	3.8/1.8	Yes	987.55	47.3	37.8	Severe	Yes	+ (3.8)	Yes	Yes	No
D‐11	Dent‐1	6.8/1	Yes	678.37	60.4	29.7	Moderate	Yes	− (6.8)	Yes	No	No
D‐12	Dent‐1	4.8/4.7	No	485.2	53.6	35	Moderate	Yes	− (4.8)	No	No	No^a^
D‐13	Dent‐1	13.5/12.3	Yes	667.57	44.4	39.8	Severe	No	− (12.3)	No	No	Yes
D‐14	Dent‐1	9.3/5.9	No	499.92	60.6	26.3	Moderate	No	NA	No	No	No
D‐15	Dent‐1	2.1/2	No	Increased	62.9	32.8	NA	Yes	+ (2.1)	No	No	No^a^
D‐16	Dent‐1	3.7/3.7	Yes	1,053.73	42.9	45.1	Severe	Yes	+ (3.7)	Yes	Yes	No
D‐17	Dent‐1	14/12	Yes	933.97	52.8	36	Severe	Yes	− (14)	No	No	No
D‐18	Dent‐1	5.4/4.8	No	688.91	61.3	24.2	Severe	Yes	− (5.4)	No	No	No
D‐19	Dent‐1	2.3/0.3	Yes	1,112.5	60.4	NA	Severe	No	− (1.9)	No	No (1.9 years)	NA
D‐20	Dent‐1	8.3/7.3	No	436.61	52.5	34.4	Moderate	Yes	− (8.3)	Yes	No	No
D‐21	Dent‐1	5.7/0.6	No	999.6	54.9	38.3	Severe	Yes	− (5.7)	No	Yes	Yes
D‐22	Dent‐1	2.3/2.1	Yes	1,091.55	53.4	38.8	Severe	Yes	+ (2.3)	No	No	No
D‐23	Dent‐1	3.7/3.6	Yes	Increased	NA	NA	NA	Yes	− (4)	No	No (4 years)	NA
D‐24	Dent‐1	10.3/7.3	No	344.9	53.4	35.7	Moderate	Yes	− (8.8)	No	No (9.3 years)	NA
D‐25	Dent‐2	15.6/0.7	No	112.55	57.6	31.9	Moderate	Yes	NA	No	No	Yes
D‐26	Dent‐2	5/3.9	No	904.63	64.4	28.4	Severe	Yes	− (4.9)	Yes	No	No
D‐27	Dent‐2	5.9/5.8	No	653.88	50.6	36	Severe	Yes	− (5.9)	No	No	No
D‐28	Dent‐2	1.6/0.2	Yes	1,918.23	51.8	34	Severe	Yes	− (1.6)	Yes	No	No
D‐29	Dent‐2	5.8/4.2	Yes	774.55	49.3	37	Severe	Yes	− (5.8)	Yes	Yes	Yes
D‐30	Dent‐2	8.1/7.2	Yes	518.09	62.6	31	Moderate	Yes	+ (8.1)	No	No	No^a^
D‐31	Dent‐2	9.5/3.8	No	567.4	39.6	47.6	Severe	Yes	NA	No	No	No^a^

Note

ACR, microalbumin‐to‐creatinine ratio; NA, not available; Percentage of LMWP and urinary albumin: analyzed by urinary protein electrophoresis; UPE, urine protein excretion; α1‐MG/Cr, urinary α1‐microglobulin‐to ‐creatinine ratio.

^a^ Renal function evaluated by Schwartz formula.

**TABLE 4:** Renal and extrarenal features of patients with Dent type 1 and type 2

	Total (*n* = 31)	Dent‐ 1 (*n* = 24)	Dent‐ 2 (*n* = 7)
Onset age (median, years)	3.7	3.4	3.9
Nephrotic‐range proteinuria	16/31	13/24	3/7
LMW proteinuria	30/30	23/23	7/7
Macroalbuminuria	18/29	13/22	5/7
Hypercalciuria	27/31	20/24	7/7
Nephrocalcinosis	8/27	7/22	1/5
Hematuria	10/31	7/24	3/7
Hypophosphatemia	5/31	4/24	1/7
Aminoaciduria	9/9	6/6	3/3
Glycosuria	3/29	3/24	0/7
Acidosis	0/31	0/24	0/7
Abnormal GFR	4/27	2/20	2/7
Short stature	9/30	4/23	5/7
Congenital cataract	3/12	1/9	2/3
DD	2/8	2/7	0/1
Rickets	4/27	4/20	0/7

Note

GFR, Glomerular filtration rate; DD, Developmental delay.

### 3.3 Extrarenal manifestations

As shown in Table 4, height was affected in 30% (9/30) of patients. Of these patients, 7 patients received steroid therapy, and 5 patients were treated with steroids for more than 6 months. Short stature was more common in patients with Dent‐2 disease. In addition to Dent‐2 patients, Dent‐1 patients also presented with congenital cataract which was diagnosed by ophthalmologist (1/9) and developmental delay (2/7). The patient with Dent‐1 disease and congenital cataract had a history of two‐week treatment with dexamethasone 10 years ago. He did not receive any corticosteroids before the diagnosis of congenital cataract was made. Rickets was detected in four patients; however, the level of serum 25‐hydroxyvitamin D in two of three patients who underwent this examination was deficient, so vitamin D deficiency associated rickets could not be excluded.

### 3.4 Renal pathology

Seventeen of 31 patients (55%) underwent a renal biopsy because of moderate to severe albuminuria or nephrotic‐range proteinuria, and 3 patients received a repeated renal biopsy. The histopathological findings are summarized in Table 5. The median interval between the age at disease onset and age at renal biopsy was 1.55 (range 0–11.5) years. Glomerular changes, including mild glomerular lesions, mesangial proliferative glomerulonephritis (MsPGN) and focal segmental glomerular sclerosis (FSGS), were found. Nine patients (53%) had mild glomerular lesions, and the median age at biopsy was 5.5 (3.7–15.4) years old, whereas the mean percentage of urinary albumin examined at the time closest to the renal biopsy was 36.9% (*n* = 5). Among them, patient 14 received a repeated renal biopsy 3.8 years later, and global sclerosis was observed in 7/21 glomeruli, which may be related to chronic tubulointerstitial nephropathy and ischemic renal damage. Patient 18 underwent a repeated renal biopsy 1.5 years later, and tubular injury with mild glomerular lesions was observed. Six patients (35%) had MsPGN, and the median age at biopsy was 6.6 (range 3.1–11) years old, whereas the mean percentage of urinary albumin analyzed at the time closest to the renal biopsy was 39.3% (*n* = 3). The remaining 2 patients (12%), who underwent a renal biopsy at the age of 13.1 and 5.8 years, respectively, had FSGS, and the percentage of urinary albumin detected at the time closest to the renal biopsy was 30.2% and 37%, respectively. In addition to FSGS, chronic tubulointerstitial damage was observed in patient 13’s repeated renal biopsy specimen (biopsy performed 2 years later).

**TABLE 5:** Pathological results of renal biopsy and changes in urinary ACR after treatment in patients with Dent disease

Pt.ID	Age at onset/renal biopsy (years)	Percentage of urinary albumin closed to renal biopsy	Pathological description	Age at treatment/length of treatment (years)	Urinary ACR (mg/g)		
					Before treatment	After treatment	Decreased percent
1	4.8/5.2	29%	MsPGN	5.3/1.7	377	192.45	49%
4	8/11	NA	MsPGN	NA	–	–	–
6	None	–	–	4.3/6.7	477.29	474.89	0.5%
7	4/15.4	36.1%	Mild glomerular lesions	NA	–	–	–
8	3/3.1	21%	MsPGN with tubulointerstitial nephritis	13.1/0.6	205.58	322.17	−57%
10	None	–	–	3.8/1.8	843.59	1,484.51	−76%
11	1/5.5	NA	MsPGN	NA	–	–	–
13	12.3/13.1	30.2%	FSGS	–	–	–	–
13	12.3/15.1	32.6%	FSGS with chronic tubulointerstitial damage	15.4/1.9	1,087.32	893.82	45%
14	5.9/7.6	NA	Mild glomerular lesions	NA	–	–	–
14	5.9/11.4	31.3%	Chronic tubulointerstitial nephropathy with ischemic renal damage	NA	–	–	–
17	12/12.1	43.3%	Mild glomerular lesions	NA	–	–	–
18	4.8/4.8	NA	Mild glomerular lesions	5.4/4.3	468.42	533	−14%
18	4.8/6.3	39.7%	Tubular injury with mild glomerular lesions	–	–	–	–
19	None	–	–	2.3/1.5	504.53	500.93	0.7%
21	0.6/5.5	38.3%	Tubular interstitial nephritis with mild glomerular lesions	NA	–	–	–
23	3.6/3.7	38.5%	Mild glomerular lesions	3.7/1.3	291.72	183.68	37%
24	7.3/8.8	NA	Mild glomerular lesions	10.7/0.3	185.5	229.84	−24%
25	0.7/11	NA	MsPGN	NA	–	–	–
26	3.9/5.1	28.4%	Mild glomerular lesions	5.7/0.4	13,302	12,874	3%
27	None	–	–	5.9/4.5	407.19	678.79	−67%
28	None	–	–	1.6/8.5	1,242	408.2	67%
29	4.2/5.8	37%	FSGS	NA	–	–	–
30	7.2/7.7	67.9%	MsPGN with tubular injury	8.1/0.3	262.96	322.95	−23%
31	3.8/5.4	NA	Mild glomerular lesions	NA	–	–	–

Note

ACR, microalbumin‐to‐creatinine ratio; MsPGN, mesangial proliferative glomerulonephritis; FSGS, focal segmental glomerular sclerosis; NA, not available.

### 3.5 Safety and the effects of ACE inhibitors and ARBs on albuminuria

**FIGURE 3:**
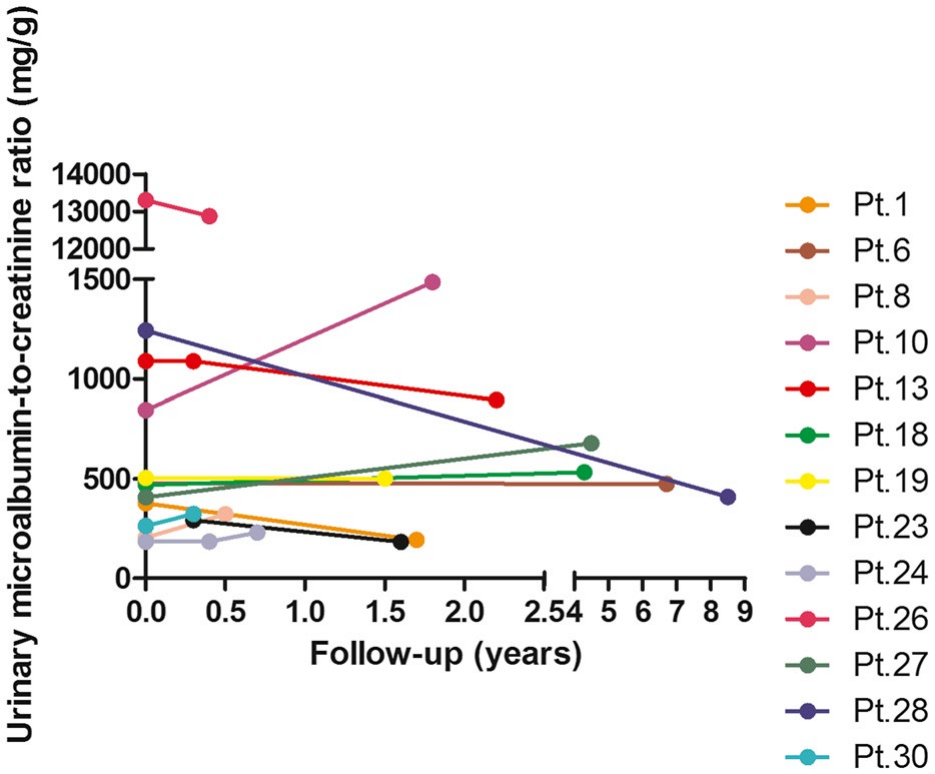
Changes in urinary ACR before and after treatment with ACE inhibitors and/or ARBs in 13 cases with Dent disease

Thirteen of 31 patients had follow‐up data and received ACE inhibitor (mainly refers to benazepril, mean dose 0.30 mg/kg/d) and/or ARB (losartan, mean dose 0.79 mg/kg/d) treatment for more than 3 months (Table 5 and Figure 3). The median age at onset of treatment was 5.4 (range 1.6–15.4) years, and the median urinary ACR before treatment was 468.42 (range 185.5–13,302) mg/g. After a median of 1.7 (range 0.3–8.5) years of treatment, a reduction in urinary ACR was observed in 54% (7/13) of children, a slight increase (less than 25%) in urinary ACR was observed in 23% (3/13), whereas a significant increase (more than 25%) was observed in the remainder. Three children (patient 10, 27 and 30) performed urinary protein electrophoresis after treatment. The proportion of urinary albumin before and after treatment were 37.8% versus 35.7%, 36% versus 39.4% and 31% versus 32.9% in these patients, respectively.

The therapy with ACE inhibitors and/or ARBs was well tolerated and did not affect renal function in 12/13 cases. However, one child stopped ARB treatment due to a decline in eGFR. One patient developed hypotension after 6 months of ACE inhibitor therapy, which resolved after taking the medication in the morning.

## 4 DISCUSSION

Male patients with a prominent manifestation of LMW proteinuria should be considered for the possibility of Dent disease, which can be diagnosed by genetic testing. However, in clinical practice, an important issue is how to think of this disease. Two clinical characteristics in this cohort are noteworthy. First, hematuria with nephrotic‐range proteinuria or moderate to severe albuminuria was common; however, it was not always associated with hypercalciuria. In the absence of a urinary erythrocyte morphology examination and urinary protein component analysis, such patients are usually recommended for renal biopsy (Nammalwar, Vijayakumar, & Prahlad, 2006); unfortunately, it is impossible to obtain a definitive diagnosis, and the patients may receive inappropriate therapy. Therefore, patients with hematuria and nephrotic‐range proteinuria or moderate to severe albuminuria should undergo an examination of urinary erythrocyte morphology and urinary protein components.

Second, patients with Dent‐1 disease may also present with congenital cataract and developmental delay. The incidence rates of cataract and intellectual impairment in Dent‐2 disease were 7% and 27%, respectively (De Matteis, Staiano, Emma, & Devuyst, 2017), whereas there was no report of these two extrarenal abnormalities in patients with Dent‐1 disease. However, in our study, patients with congenital cataract or developmental delay had been observed in Dent‐1 disease. A study showed CLC proteins (including CLC‐5 encoded by the *CLCN5* gene) could regulate electrical excitability of neurons (Mufson et al., 2018), thus, the relationship between developmental delay and *CLCN5* mutations may exist. Of course, these abnormalities may also coexist with Dent‐1 disease, whereas they need to be differentiated from Lowe syndrome and Dent‐2 disease. Thus, patients with suspected Dent disease should undergo ophthalmological examination and intelligence assessment.

Another interesting aspect of the present study is the opportunity to evaluate the percentage of urinary albumin and the effects of ACE inhibitor and/or ARB therapy on albuminuria. The percentage of urinary albumin in the total protein ranged from 24.2% to 47.6%; it was usually smaller than that of LMW proteinuria. The use of ACE inhibitors and/or ARBs effectively decreased the albuminuria in half of the children with glomerular pathology, including FSGS, mild glomerular lesions and MsPGN, and this therapy was well tolerated. A similar promising reduction in urine total protein was observed in two boys with less than 1 year of treatment, and the histologic findings were FSGS and extensive mesangial deposition of C1q (Copelovitch, Nash, & Kaplan, 2007; Lim, Yun, Moon, & Cheong, 2007). However, it has also been reported that the level of urine total protein in some patients failed to respond to ACE inhibitor and/or ARB therapy after a maximum of 5 years (Blanchard et al., 2016; Cramer et al., 2014; Frishberg et al., 2009; Vaisbich et al., 2012); unfortunately, data on the efficacy of these drugs on albuminuria were not available. Our results showed that, in half of the children, albuminuria could be controlled with ACE inhibitor and/or ARB treatment. Genotypes, glomerular lesions, the age at the initiation of treatment and the duration of treatment could not explain the difference in antialbuminuric response to ACE inhibitor and/or ARB therapy. In the future, it is worth observing whether patients with Dent disease benefit from long‐term albuminuria control.

There are several limitations to this study. First, this was a retrospective study with incomplete medical examinations; for example, not all patients underwent urinary amino acid screening, intelligence assessment and ophthalmological examination. Baseline data of some patients were missing. The second limitation is the absence of urinary protein electrophoresis in most patients during follow‐up, and the proportion of urinary albumin after treatment was unknown. The third limitation is the low precision of estimating GFR using 24‐hr Ccr and the Schwartz formula (Wang et al., 2010). A further limitation was the short follow‐up time, and the long‐term effects of ACE inhibitor/ARB therapy are not clear.

In conclusion, hematuria with nephrotic‐range proteinuria or moderate to severe albuminuria was common in Dent disease patients. Extrarenal manifestations could be observed in a small number of Dent‐1 patients, which extends the phenotypic spectrum of the disease. In addition, ACE inhibitors/ARBs are well tolerated, and they are partially efficient in controlling albuminuria in Dent disease.

## CONFLICT OF INTEREST

None declared.

## Data Availability

All data analyzed during this study are included in this article.
